# Modified early obstetric warning scores: A promising tool but more evidence and standardization is required

**DOI:** 10.1111/aogs.13448

**Published:** 2018-10-16

**Authors:** Tanya Robbins, Andrew Shennan, Jane Sandall

**Affiliations:** ^1^ Department of Women and Children’s Health King’s College London London UK

**Keywords:** early warning system, morbidity, mortality, pregnancy

## Abstract

Early warning systems involve the routine monitoring and recording of vital signs or clinical observations on specifically designed charts with linked escalation protocols. Meeting criteria for abnormal physiological parameters triggers a color‐coded or weighted scoring system aimed to guide the frequency of monitoring, need for, and urgency of clinical review. Color‐coded systems trigger a clinical response when one or more abnormal observation is recorded in the red zone or two or more mildly abnormal parameters in the amber zone. The principle of maternity‐specific early warning systems to structure surveillance for hospitalized women is intuitive. The widespread use and policy support, including recommendations following confidential enquiries and from the National Health Service Litigation Authority, is not, however, currently backed up by a strong evidence base. Research is required to develop predictive models and validate evidence‐based maternity‐specific early warning systems in the general maternity population.

**Abbreviations**
CI: confidence interval
EWS: early warning system
ICU: intensive care unit
MEOWS: modified early obstetric warning score

**Key message**

Current MEOWS trigger thresholds vary, based arbitrarily on clinical consensus or expert opinion. Limited evidence links implementation of MEOWS with improved outcomes in the general pregnant population and further research is required to develop predictive models and validate evidence‐based MEOWS.

## 1 INTRODUCTION

Maternal deaths in the UK are rare (4.65 per 100 000 live births in 2012‐2014) compared with the global burden (216 per 1 00 000 live births in 2015).^1^, ^2^ 99% of maternal mortalities occur in low‐income and middle‐income countries. Many women die from preventable causes. Despite a 44% reduction in maternal deaths globally between 1990 and 2015, approximately 830 women still die every day. There has been no significant change in the maternal death rates in the UK between 2009‐2011 and 2012‐2014, although there is a downward trend. The women whose deaths were reported in the last UK triennial report left behind 358 children.

Delay is a major contributor to maternal mortality. The effective management of an acutely unwell pregnant woman can be challenging and requires an appropriate and timely response. An approach to tackling this clinical challenge is the use of early warning systems (EWS). EWS involve the routine monitoring and recording of vital signs or clinical observations on specifically designed charts with linked escalation protocols. They list criteria for abnormal physiological parameters that trigger a color‐coded or weighted scoring system aimed to guide the frequency of monitoring, need for, and urgency of clinical review. Color‐coded systems trigger a clinical response when one or more abnormal observation is recorded in the red zone or two or more mildly abnormal parameters in the amber zone. In this commentary we will examine the evidence base behind modified early obstetric warning scores (MEOWS) and discuss the issues surrounding current practice.

The UK Confidential Enquiry into Maternal Deaths published in 2007 recommended the introduction of MEOWS for all acute obstetric admissions, including women in early pregnancy.^3^ In 2012 the Royal College of Physicians introduced national early warning scores for use in the general adult population.^4^ Thereafter, there has been a growing trend towards the use of similar charts in the pregnant population. The MEOWS included in the 2007 UK Confidential Enquiry consisted of scores of respiratory rate, oxygen saturation, temperature, heart rate, blood pressure, assessment of urine, including for proteinuria, color of amniotic fluid, neurological response, pain score, assessment of lochia, and an overall assessment of whether the woman appears well.^3^

Subsequent reports have recommended their use but have also acknowledged their shortcomings and the lack of an evidence‐based standardized approach in maternity populations. The latest report published in 2016 raises the issue of ensuring that MEOWS remain fit for purpose in view of developments in modern information technology, near patients’ testing, and sepsis red flags.^1^

A principal component of an EWS relies on a phase of decompensation in the physiological parameters measured; signaling an opportunity to intervene and prevent further deterioration. Concerns have been raised in translating this principle directly to the pregnant population with its unique physiological adaptations, which may allow for a longer period of apparent compensation but one that can be followed by an abrupt deterioration. For this reason the level of consciousness as a parameter in an early warning score has been criticized. A reduced or altered level of consciousness is not an early warning sign but a red flag indicating established illness.^1^ Further, there is a paucity of data from the low‐risk pregnant population needed to establish the normal distributions of parameters for gestation‐specific vital signs. The Oxford‐based Pregnancy Physiology Pattern Prediction study, currently in progress, aims to collect these data and will provide a valuable basis for the development of a national obstetric EWS.^5^ It may assist in addressing the question of whether gestation‐specific MEOWS are necessary. However, it may not provide an answer as to how feasible this system may be or which vital signs or combinations are most predictive of maternal deterioration.

## 2 WHAT IS THE CURRENT EVIDENCE?

A recent analysis of charts of vital signs from consultant‐led maternity units across the UK demonstrated considerable variation in EWS and escalation protocols.^6^ Smith et al extracted data from 120 such units and found they included a wide range of supposedly normal vital signs. In some units these parameters overlapped with those used to identify sepsis, a leading cause of maternal morbidity and mortality, as well as mild diastolic and severe systolic hypertension, as defined by the National Institute for Health and Care Excellence. A variation in escalation protocols was also noted in the use of a color‐coded trigger system, an aggregate weighted scoring system or a combination of both.

Bick et al performed a cross‐sectional survey across the maternity services of the UK National Health Service.^7^ All except one of the 108 organizations that responded reported using EWS. Of these, 99% used them antenatally, 76% for women in labor, and 100% for postnatal. The challenges reported included overlapping EWS with the use of a partogram in labor, staff shortages, and delays in obtaining clinical reviews when escalation was required.

A systematic review conducted to aid in the development of an Irish national guideline looked at 33 studies separated into four categories—descriptive studies, clinical practice guidelines, effectiveness studies, and development and validation studies.^8^ They found little high‐quality evidence that MEOWS were being developed and tested for their predictive ability. The evidence currently available focuses on selected high‐risk obstetric populations. This review found that compliance with recording observations varied and was related to training issues. It improved after audit and diminished with the increasing length of inpatient stay.

An ethnographic study conducted in two UK hospitals over a 7‐month period also found that charts were used at the health provider’s discretion.^9^ The study, included in the systematic review, reported that MEOWS charts were perceived by some staff members as a challenge to their clinical judgement. Staff members, particularly in postnatal wards, raised concerns over what they perceived as the medicalization of healthy women, and the impact that the universal use of MEOWS would have on care, such as support for breastfeeding. There is a particular need to demonstrate their benefit with appropriate professional education in low‐risk women. Charts may have their highest impact in a population where clinical concerns are least, such as on the postnatal ward. It was reported that the positive aspects of MEOWS charts include their use as a visual aid to recognize negative trends especially where the decline is gradual. Defined thresholds enable staff to identify deviations from them and legitimize summoning help across hierarchical boundaries. Accordingly, these charts were seen in some cases as useful in escalating care quickly. They may also increase team awareness of critical situations.

A validation study looking prospectively at 676 consecutive maternity inpatient admissions reviewed MEOWS triggers and morbidity.^10^ The MEOWS chart used was adapted from the 7^th^ CEMACE report. Outcomes at 30 days were retrieved from hospital records and patients’ notes including morbidity, death, admission to ICU, and discharge. The data analyzed were tested for associations between abnormal triggers and the presence of morbidity by calculating the relative risk for individual parameters. They report 89% sensitivity (95% CI 81%‐95%) and 79% specificity (95% CI 76%‐82%) and concluded that MEOWS charts are a useful bedside test for predicting morbidity. A low positive predictive value of 39% indicated a low prevalence of maternal morbidity. The authors of this study conclude that this supports the widespread use of MEOWS for all obstetric patients. Although MEOW’s ability to identify morbidity is promising, further evidence is needed to demonstrate the benefit of introducing this to the pregnant population as a whole. This would include its benefit over established clinical practice and health economic benefits.

A retrospective case‐controlled study carried out across 2 tertiary obstetric units in Canada extracted MEOWS variables in the 24 h prior to women’s admission to the ICU.^11^ This validation study found MEOWS to be very sensitive in predicting ICU admission and reported it had a high negative predictive value. However, MEOWS was not specific, and many controls who had no complications also triggered on thresholds used. Four variables (maximum temperature, heart rate, systolic blood pressure, and respiratory rate) were significantly associated with admission to the ICU. Secondary analyses and the use of the four variable model had high sensitivity, specificity, negative predictive value, and area under the receiver operating characteristic curve for admission to the ICU.

Shields et al conducted a pilot study over 6 sites in the USA following the implementation of a maternity early warning trigger tool.^12^ They aimed to evaluate prospectively the use of a pathway‐specific early warning trigger tool and determine whether it was associated with reduced maternal morbidity compared with controls in non‐pilot sites. The tool addressed four main causes of morbidity—sepsis, cardiovascular dysfunction, severe preeclampsia or hypertension, and severe hemorrhage, and included recommendations for patients’ assessment and treatment. The tool had two levels of activation—non‐severe, which required two triggers, or severe, requiring a single abnormal trigger. They reported a significant reduction in Centers for Disease Control‐defined severe maternal morbidity (*P* = 0.01) and composite maternal morbidity (*P* = 0.01) comparing baseline and after the implementation of the maternity early warning trigger tool. The authors suggest that areas to focus on in the development of an EWS are ease of use and an alert frequency low enough to prevent alarm fatigue but high enough for clinicians to associate value with the trigger.

Carle et al performed a retrospective secondary analysis on physiological data collected for a routine audit from a critical care obstetric population.^13^ They set out to develop (n = 2240) and internally validate (n = 2200) an obstetric early warning system using separate samples of women. The most abnormal measurements within the first 24 h following admission to critical care were analyzed. Significant variables (*P* < 0.05) were weighted following multiple logistic regressions and used to create a statistical obstetric trigger system. The primary outcome was survival. They reported an area under the receiver operator curve of 0.995 (95% CI 0.992‐0.998) for the resulting statistical score. Additional variables were then added to create a clinical score. This included high systolic and diastolic blood pressure and temperature >38°C, although these variable were not demonstrated to be statistically significant. Their addition to this score was felt necessary to make the system clinically relevant and acceptable and did not result in a significant decrease in the score’s discriminatory ability. A sensitivity of 97% and a specificity of 87% were quoted with a score of 12. This may indicate it is able to discriminate between survivors and non‐survivors but it does not validate a system for the early detection of deterioration. Although this article reports on a large data set, it cannot be directly translated to the ward setting and further external validation is required. Furthermore, a retrospective secondary analysis of data has potentially missed clinically relevant data. The non‐ventilated respiratory rate was absent from 96% of the cases that resulted in death in the validation set of data.

## 3 DISCUSSION

The principle of a maternity‐specific EWS to structure surveillance for hospitalized women is intuitively sound. Their current widespread use and policy support, including recommendations following confidential enquiries and from the National Health Service Litigation Authority, are not, however, currently backed by a strong evidence base. There is limited evidence linking the implementation of MEOWS charts to improved outcomes in the general pregnant population. Deaths in the UK obstetric population are rare and the challenge is to equate the impact of MEOWS with meaningful outcomes. There appears to be room for improvement as substandard care continues to be highlighted in recurrent confidential enquiries.^1^ Future research aiming to identify the components of MEOWS that are most strongly related to adverse outcomes and their relative interdependence on accompanying implementation outcomes may help to simplify management strategies and escalation protocols. The UK Royal College of Anaesthetists has recommended only one intermediate step prior to review by a senior clinician.^14^

The wide variation that exists in chart layout and implementation undermines confidence in the validity of MEOWS.^6^ Current trigger thresholds vary and are arbitrarily based on clinical consensus or expert opinion. Additional research is required to develop predictive models and to validate MEOWS in a general obstetric population, preferably with well‐designed controlled studies and appropriate health economics. Future studies could have a global focus, including the collection of clinical variables in low‐resource settings where intervention bias is less likely and adverse outcomes are more common. The issues of compliance in monitoring and recording may be addressed with the introduction of automated monitoring and trigger devices. The benefits of an automated EWS include improving the audit trail, the environmental benefits of a paperless system, time efficiency, and improved consistency. However, MEOWS should not be considered a substitute for clinical evaluation and assessment.

## CONFLICT OF INTEREST

AS has developed an automated vital signs monitoring device (CRADLE Vital Signs Alert) with Microlife, incorporating a traffic light triage system. AS is principle investigator on the ongoing CRADLE Projects. JS is co‐investigator on the CRADLE 3 trial.

## References

[1] Knight M , Nair M , Tuffnell D , On behalf of MBRRACEUK. Surveillance of Maternal Deaths in the UK 2012–14 and Lessons Learned to Inform Maternity Care from the Uk and Ireland Confidential Enquiries into Maternal Deaths and Morbidity 2009–14. Oxford, UK: MBRRACE; 2016.

[2] WHO, UNICEF, UNFPA, World Bank Group and the United Nations Population Division . Trends in Maternal Mortality 1990–2015. Geneva, Switzerland: WHO.

[3] Lewis G , Clutton‐Brock T , Cooper G, et al. Saving Mothers' Lives: Reviewing maternal Deaths to Make Motherhood Safer 2003‐2005. London, UK: CEMACH; 2007.

[4] Royal College of Physicians . National Early Warning Score (News): Standardising the Assessment of Acute‐Illness Severity in the NHS. London, UK: RCP; 2012.

[5] Kumar F , Kemp J , Edwards C , et al. Pregnancy physiology pattern prediction study (4P study): protocol of an observational cohort study collecting vital sign information to inform the development of an accurate centile‐based obstetric early warning score. BMJ Open. 2017;7:e016034. 10.1136/bmjopen-2017-016034 PMC5589023 28864695

[6] Smith GB , Isaacs R , Andrews L , et al. Vital signs and other observations used to detect deterioration in pregnant women: an analysis of vital sign charts in consultant‐led UK maternity units. Int J Obstet Anesth. 2017;30:40‐51. 10.1016/j.ijoa.2017.03.002 28385419

[7] Bick DE , Sandall J , Furuta M , et al. A national cross sectional survey of heads of midwifery services of uptake, benefits and barriers to use of obstetric early warning systems (EWS) by midwives. Midwifery. 2014;30:1140‐1146. 24820002 10.1016/j.midw.2014.03.016

[8] Devane D , Clark M , Sandall J , et al. Early Warning Scores in Maternity Care: A Systematic Review. Dublin, Ireland: National Clinical Effectiveness Committee; 2014.

[9] Mackintosh N , Watson K , Rance S , Sandall J . Value of a modified early obstetric warning system (MEOWS) in managing maternal complications in the peripartum period: an ethnographic study. BMJ Qual Saf. 2014;23:26‐34. 10.1136/bmjqs-2012-001781 23868867

[10] Singh S , McGlennan A , England A , Simons R . A validation study of the CEMACH recommended modified early obstetrics warning system (MEOWS). Anaesthesia. 2012;67:12‐18. 22066604 10.1111/j.1365-2044.2011.06896.x

[11] Ryan HM , Jones MA , Payne BA , et al. Validating the performance of the modified early obstetric warning system multivariable model to predict maternal intensive care unit admission. J Obstet Gynaecol Can. 2017;39:728‐733. 28566256 10.1016/j.jogc.2017.01.028

[12] Shields LE , Wiesner S , Klein C , Pelletreau B , Hedriana , et al. Use of maternal early warning trigger tool reduces maternal morbidity. Am J Obstet Gynecol. 2016;214:527. 26924745 10.1016/j.ajog.2016.01.154

[13] Carle C , Alexander P , Columb M , Johal J . Design and internal validation of an obstetric early warning score: secondary analysis of the Intensive Care National Audit and Research Center Case Mix Programme database. Anesthesia. 2013;68:354‐367. 10.1111/anae.12180 23488833

[14] Royal College of Anaesthetists . Care of the Critically Ill Woman in Childbirth; Enhanced Maternal Care. London, UK: RCoA, RCOG, RCM, ICS, FICM and OAA; 2018.

